# A Facile, Fabric Compatible, and Flexible Borophene Nanocomposites for Self‐Powered Smart Assistive and Wound Healing Applications

**DOI:** 10.1002/advs.202201507

**Published:** 2022-06-03

**Authors:** Shuo‐Wen Chen, Shih‐Min Huang, Han‐Song Wu, Wei‐Pang Pan, Shih‐Min Wei, Chih‐Wei Peng, I‐Chih Ni, Bayu Tri Murti, Meng‐Lin Tsai, Chih‐I Wu, Po‐Kang Yang

**Affiliations:** ^1^ Department of Biomedical Sciences and Engineering National Central University Taoyuan 32001 Taiwan; ^2^ Department of Materials Science and Engineering National Taiwan University of Science and Technology Taipei 10607 Taiwan; ^3^ School of Biomedical Engineering College of Biomedical Engineering Taipei Medical University Taipei 11031 Taiwan; ^4^ Institute of Photonics and Optoelectronics and Department of Electrical Engineering National Taiwan University Taipei 10617 Taiwan; ^5^ Graduate Institute of Biomedical Materials and Tissue Engineering Taipei Medical University Taipei 11031 Taiwan

**Keywords:** borophene, medical assistive, nanocomposite, triboelectricity, wound healing

## Abstract

Smart fabrics that can harvest ambient energy and provide diverse sensing functionality via triboelectric effects have evoked great interest for next‐generation healthcare electronics. Herein, a novel borophene/ecoflex nanocomposite is developed as a promising triboelectric material with tailorability, durability, mechanical stability, and flexibility. The addition of borophene nanosheets enables the borophene/ecoflex nanocomposite to exhibit tunable surface triboelectricity investigated by Kelvin probe force microscopy. The borophene/ecoflex nanocomposite is further fabricated into a fabric‐based triboelectric nanogenerator (B‐TENG) for mechanical energy harvesting, medical assistive system, and wound healing applications. The durability of B‐TENG provides consistent output performance even after severe deformation treatments, such as folding, stretching, twisting, and washing procedures. Moreover, the B‐TENG is integrated into a smart keyboard configuration combined with a robotic system to perform an upper‐limb medical assistive interface. Furthermore, the B‐TENG is also applied as an active gait phase sensing system for instantaneous lower‐limb gait phase visualization. Most importantly, the B‐TENG can be regarded as a self‐powered in vitro electrical stimulation device to conduct continuous wound monitoring and therapy. The as‐designed B‐TENG not only demonstrates great potential for multifunctional self‐powered healthcare sensors, but also for the promising advancements toward wearable medical assistive and therapeutic systems.

## Graphical abstract

The development of novel borophene/ecoflex nanocomposite as a new triboelectric layer with excellent stability, flexibility, durability, and triboelectricity is demonstrated. The addition of borophene nanosheets strongly enhances the triboelectric charge transfer process. The borophene/ecoflex is further integrated into a B‐TENG for biomechanical energy harvesting, biomedical assistive platform, and wound healing applications.

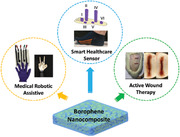

## 1 Introduction

Owing to the rapid development of wearable technologies, numbers of electronic products are currently evoked, such as wearable patches,^[^ ^1^ ^]^ electronic clothing,^[^ ^2^, ^3^ ^]^ biomedical sensors,^[^ ^4^ ^]^ and human–machine interfaces.^[^ ^5^, ^6^, ^7^ ^]^ However, how to properly power these wearables still remains a critical challenge. Triboelectric nanogenerators (TENGs), which transfer ambient mechanical energy into electricity have been widely investigated for their power sustainability, material accessibility, textile compatibility, and diverse applicability to wearable electronics.^[^ ^8^, ^9^, ^10^ ^]^ Specifically, the development of novel nanocomposite triboelectric material has been regarded as a promising approach to enhance the feasibility of utilizing TENGs to power commercial electronics.

2D materials, which possess unique properties within atomic scale, have been reported as the candidates for nanocomposite formation in TENG design.^[^ ^11^, ^12^, ^13^, ^14^, ^15^ ^]^ For example, Bayan et al. reported a photo‐active TENG based on a few‐layer carbon nitride nanosheets (g‐C_3_N_4_ NSs), where g‐C_3_N_4_ served as a novel tribo‐layer to enhance the output performance.^[^ ^12^ ^]^ Wu et al. reported an enhanced output performance of TENGs achieved by inserting molybdenum disulfide nanosheets (MoS_2_ NSs) as an electron acceptor layer.^[^ ^13^ ^]^ The output enhancement can be attributed to the effective charge trapping phenomenon to reduce the charge loss in the contact–separation process. Xiong et al. also reported that black phosphorous nanosheets (BP NSs) with high specific surface area and quantum‐confinement effect can significantly promote the output performance of TENGs.^[^ ^14^ ^]^ Recently, borophene, containing layered‐by‐layered boron atoms with structural complexity, high anisotropy, electronic transport, and mechanical properties, has received great attention in various kinds of technological aspects.^[^ ^15^, ^16^, ^17^ ^]^ However, only few studies have been found to access the triboelectric properties of borophene.

TENGs have been widely applied to healthcare applications, such as gait phase monitoring,^[^ ^18^ ^]^ wearable assistive,^[^ ^19^ ^]^ in vitro pacemaker,^[^ ^20^ ^]^ and electronic skins.^[^ ^21^ ^]^ However, previous attempts mostly focus on physically healthy users, while they may become inactive or disabled under specific conditions. Additionally, it is noteworthy that wound healing acceleration from real‐time electrical stimulation (ES) provided by TENGs also made a great impact on human‐centric therapeutics, which further enable TENGs to possess therapeutic functionality very recently.^[^ ^22^ ^]^ Nevertheless, conventional strategies of using TENGs in wound healing applications target the recovery of incision wounds because of their higher probability to be invaded and infected by the ambience.^[^ ^23^, ^24^, ^25^ ^]^ In contrast, bruises and pressure sores caused by impact or pressure are also injuries that need to be paid more attention, especially for long‐term bedridden patients and people with disabilities who cannot move by themselves.

Herein, we report a facile, fabric‐compatible, and flexible B‐TENG based on the borophene/ecoflex nanocomposite for energy harvesting, medical assistive, and wound healing applications. The borophene/ecoflex nanocomposite possesses high surface charge density, cost‐effectiveness, large scalability, flexibility, and durability. The addition of borophene nanosheets (NSs) enable the nanocomposite layer with tunable surface triboelectricity investigated by Kelvin probe force microscopy (KPFM). Additionally, B‐TENG is integrated into a smart keyboard configuration and combined with the robotic system to form an upper‐limb medical‐assistive interface toward the users with disability. Moreover, B‐TENG can also serve as an active sensing unit for lower‐limb gait phase visualization platform design. Most importantly, we demonstrate the feasibility of using B‐TENG in providing ES for wound therapy, where in vitro cellular behavior and corresponded animal model were implemented. We believe that as‐designed B‐TENG will not only shed light on advanced self‐powered sensing systems, but also pave the way for future medical‐assistive technology and wearable therapeutics.

## 2 Results and Discussion

**Figure 1:**
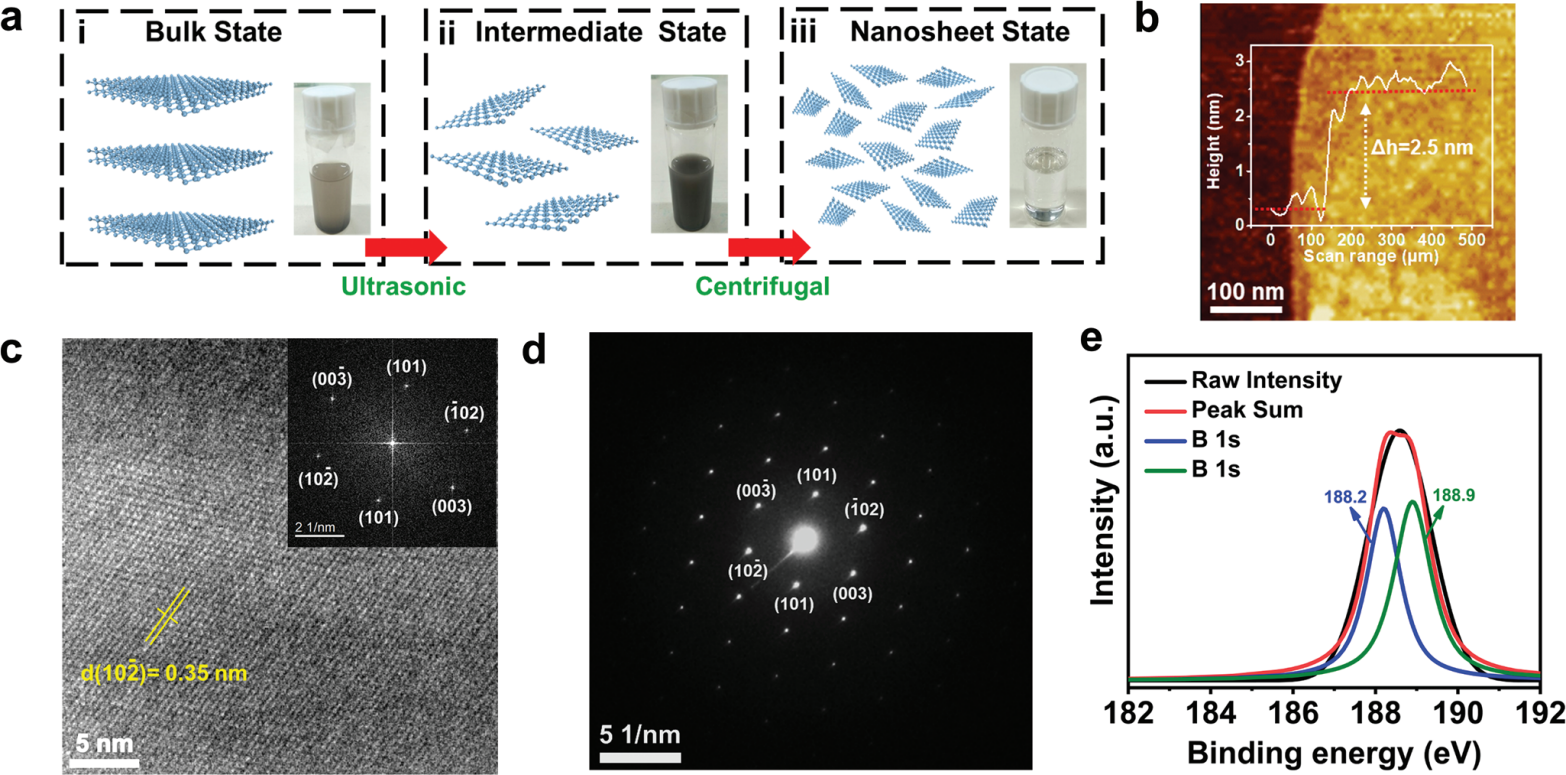
a) A schematic process to produce borophene NSs. b) AFM image of a single borophene NS. c) HR‐TEM image of a single borophene NS. The FFT pattern is shown as the inset. d) Corresponded SAED pattern. e) XPS spectrum of a single borophene NS.

The borophene NSs are derived from the liquid‐phase exfoliation method that has been previously reported.^[^ ^26^ ^]^ **Figure** **1**a shows a schematic fabrication process of borophene NSs with the molecular structure, where three stages with proper ultrasonication and suspension control, including i) bulk state, ii) intermediate state, and iii) nanosheet state, are presented. Figure 1b shows atomic force microscopy (AFM) image of borophene, which exhibits a sheet‐like structure with thickness around 2.5 nm, which can be estimated to be five to six layers in accordance with the ≈4.16 Å of monolayer borophene.^[^ ^27^ ^]^ The lattice structure and atomic‐scale arrangement of borophene NSs were characterized by using high‐resolution transmission electron microscopy (HR‐TEM). Figure 1c shows the HR‐TEM image of borophene NS with an interplanar spacing of $d_{\left(10\bar{2}\right)}$= 0.35 nm belonging to rhombohedral *α*‐phase boron phase refer to JCPDS PDF #78‐1571.^[^ ^28^, ^29^ ^]^ The corresponding fast Fourier transform (FFT) pattern is also shown as the inset in Figure 1c. The selected‐area electron diffraction (SAED) pattern of borophene NS along the [010] zone‐axis is shown in Figure 1d. In Figure 1e, X‐ray photoelectron spectroscopy (XPS) was applied to analyze the chemical composition of as‐derived borophene NS. It can be observed that two deconvoluted peaks appear with the binding energy of 188.2 and 188.9 eV, which correspond to the characteristic B 1s orbital signal of borophene.^[^ ^30^ ^]^ The wide survey scan for borophene is also shown in Figure S1 in the Supporting Information.

**Figure 2:**
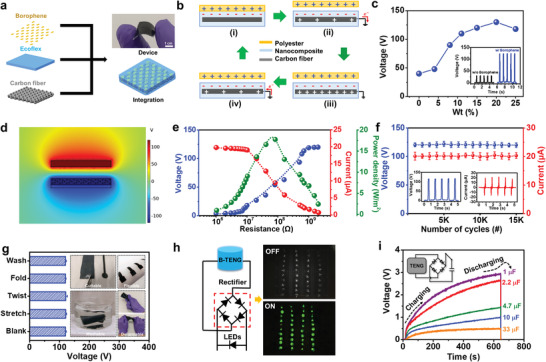
a) A schematic integration process of producing B‐TENG. b) Schematic illustration of the working mechanism of B‐TENG. c) Output voltage of B‐TENG with different borophene concentrations. The inset shows the output comparison between optimal (20 wt%) and pristine device (0 wt%). d) Output voltage distribution of B‐TENG by COMSOL simulation. e) Dependence of current, voltage, and instantaneous power density of B‐TENG with different external loads. f) Stability tests of output voltage and current of B‐TENG. g) Output voltage of B‐TENG after folding, stretching, twisting, and severe washing treatments. The inset photos are the corresponding images of various treatments. h) 40 LEDs can be lit up by B‐TENG. i) The stored charge–time relationship of B‐TENG with different capacitors.

A schematic process of fabricating borophene/ecoflex nanocomposite (Ecoflex‐B) is shown in **Figure** **2**a. The Ecoflex‐B consists of a layer of ecoflex, borophene NS, and carbon fiber (CF), where the CF serves as the flexible electrode material. The details of fabricating Ecoflex‐B are described in the Experimental Section. The field‐emission scanning electron microscope images of Ecoflex‐B are also shown in Figure S2 in the Supporting Information. The as‐fabricated Ecoflex‐B was further combined with a nylon‐coated fabric to form a B‐TENG. Figure 2b illustrates the working mechanism of B‐TENG. There are four stages within the contact separation process, denoted as (i) to (iv). When the polyester contacts with Ecoflex‐B, charges would be transferred from the polyester to Ecoflex‐B due to a higher surface electron affinity. When the polyester separates from Ecoflex‐B, the negative charges will induce positive charges on CF to compensate the triboelectric charges, causing the electrons to flow from the CF to the ground. This process generates an output signal. When the negative triboelectric charges are balanced by the induced positive charges, no output signals are detected. When the polyester approaches Ecoflex‐B, the positive charges on CF will decrease, causing the electrons flow from the ground to CF until the polyester and Ecoflex‐B become fully in contact again, causing a reversed output signal. The corresponding simulated electric potential distribution of B‐TENG within the operation process was investigated by using COMSOL Multiphysics software, as shown in Figure S3 in the Supporting Information.

Optimization of mixture conditions is the key to achieving reliable output performance of nanocomposite‐based TENGs.^[^ ^31^ ^]^ Herein, the output performances of B‐TENG with different mixture conditions are investigated in Figure 2c and Figure S4 in the Supporting Information. It can be found that the output performance of B‐TENG increases with higher concentration of borophene NSs. The maximum output performance was obtained at 20 wt%, where the output voltage and the current were up to 120 V and 20 µA in comparison with the pristine device. The enhancement of output performance could be correlated with the addition of borophene NSs regarded as an electron acceptor medium.^[^ ^32^ ^]^ To further clarify the effect of borophene addition on output performance of B‐TENG, the distribution of electric potential between two tribo‐interfaces inside B‐TENG was investigated by using COMSOL Multiphysics software in Figure 2d and Figure S5 in the Supporting Information. It is found that the simulation results were in accordance with the output performance in real devices. Figure 2e displays the output power density measured by loading different external resistances. The instantaneous power density can reach 18 W m^–2^ with external load of 20 MΩ. Figure 2f demonstrates the stability test of B‐TENG. The results indicate that the B‐TENG was highly durable and reliable even up to 10 000 testing cycles. Figure 2g demonstrates the durability of B‐TENG. The corresponded output performance was evaluated after being subjected to 500 cycles of folding, twisting, stretching, and severe washing treatments for 24 h, as shown in the inset of Figure 2g. The results indicate the outstanding durability of the as‐fabricated B‐TENG. The tailorability of B‐TENG is also investigated in Figure S6 in the Supporting Information. Moreover, the dependence of operation frequencies of B‐TENG is evaluated in Figure S7 in the Supporting Information. It can be seen that the B‐TENG can harvest ambient mechanical energy with various operation frequencies from 0.5 to 2.5 Hz. The dependence of output performance of B‐TENG on applied pressure is also presented in Figure S8 in the Supporting Information.

To understand the feasibility of using B‐TENG as a power source for commercial electronics, the B‐TENG was connected to a full‐wave rectifying bridge, and further applied to drive commercial light‐emitting diodes (LEDs) and capacitors. In Figure 2h, one can see that 40 LEDs can be successfully driven by B‐TENG. In Figure 2i, the B‐TENG can charge the capacitors with different nominal capacitances, which range from 1 to 33 µF. It can be observed that the charging characteristics gradually increased and finally reached a saturation state. The results demonstrate that B‐TENG was able to power commercial electronics. The comparison of using B‐TENG and pristine devices to charge commercial capacitors (2.2 µF) is also shown in Figure S9 in the Supporting Information.

**Figure 3:**
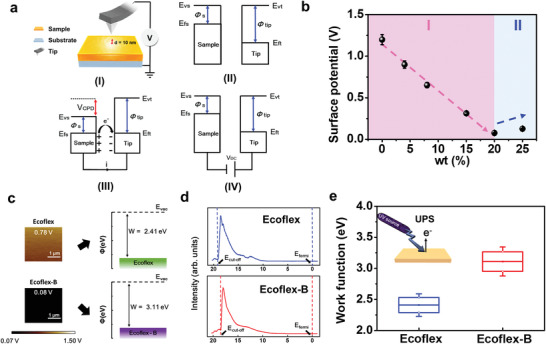
a) Schematic diagram of measuring process in KPFM. (I–IV) depict the corresponding changes of band diagram within the measuring process. b) Correlation between surface potential and concentration of borophene addition. c) Surface potential images and corresponded work function of Ecoflex and Ecoflex‐B (20 wt%). d) UPS spectra of Ecoflex and Ecoflex‐B. e) The work function comparison from UPS between Ecoflex and Ecoflex‐B. The inset image is a schematic system setup of UPS measurement.

KPFM has been widely applied to investigate the distribution of surface charge within triboelectric interface which is strongly correlated with the output performance of TENG.^[^ ^33^ ^]^ Herein, the surface potential can be defined as the voltage difference between the tip and the sample. The voltage (*V*_CPD_) is applied to eliminate the contact potential difference (CPD), as shown in **Figure** **3**a. The formula of work function is defined as below

(1) $$\phi_{\mathrm{sample}}=\phi_{\mathrm{tip}}-eV_{\mathrm{CPD}}$$

where *ϕ*_tip_ is the work function of the tip, *V*_CPD_ is the value of CPD between tip and sample, and *e* is the charge of electron. The measuring process is described as follows. First, the initial energy levels of the tip and sample are shown in Figure 3a‐II. As the tip gets closer to the sample surface, an electron transfer process occurs between two interfaces, leading to the alignment of Fermi levels of tip and sample. A steady‐state equilibrium is thus achieved, as shown in Figure 3a‐III. Finally, an external voltage (*V*_DC_) is applied to the tip to neutralize the charge of electrons induced by *V*_CPD_, as shown in Figure 3a‐IV. In Figure 3b, the surface potential of Ecoflex‐B with different mixing conditions was explored. The surface potential will keep decreasing until the mixing condition reached 20 wt%. The Ecoflex‐B exhibits a more negative value in surface potential as compared to Ecoflex (light pink region). The results indicate that the addition of borophene NSs could serve as an electron trapping layer to enhance the output performance of B‐TENG. Meanwhile, the surface potential of Ecoflex‐B is found to be slightly increased after 20 wt% (light blue region). The results may be attributed to the localized aggregation of borophene NSs that reduce the effective contacting area to generate triboelectric outputs.^[^ ^34^ ^]^ In Figure 3c, the surface potentials were measured as 0.78 V for Ecoflex and 0.08 V for Ecoflex‐B, respectively. The corresponded work function was 2.41 and 3.11 eV for Ecoflex and Ecoflex‐B estimated via formula (1).

Ultraviolet photoelectron spectroscopy (UPS) is a useful tool for the identification of work function.^[^ ^35^ ^]^ The work function can be derived from the formula (*E*_W.F_ = *hv* − (*E*_cutoff_ − *E*_F_)), where the excitation source energy (HeI, *hv* = 21.22 eV). The UPS measurement was conducted on both Ecoflex and Ecoflex‐B, as shown in Figure 3d. On the basis of the results from UPS measurement, the work functions were calculated ≈2.41 (± 0.12) and 3.11 (± 0.16) eV for Ecoflex and Ecoflex‐B, as shown in Figure 3e, which are well‐matched with KPFM analysis (Figure 3c). As discussed above, it is expected that the electron transfer from the polyester to Ecoflex‐B is more effective than Ecoflex. More induced charges will maintain on top of the surfaces in Ecoflex‐B, which can lead to the enhancement in output performance of B‐TENG (inset of Figure 2c).

**Figure 4:**
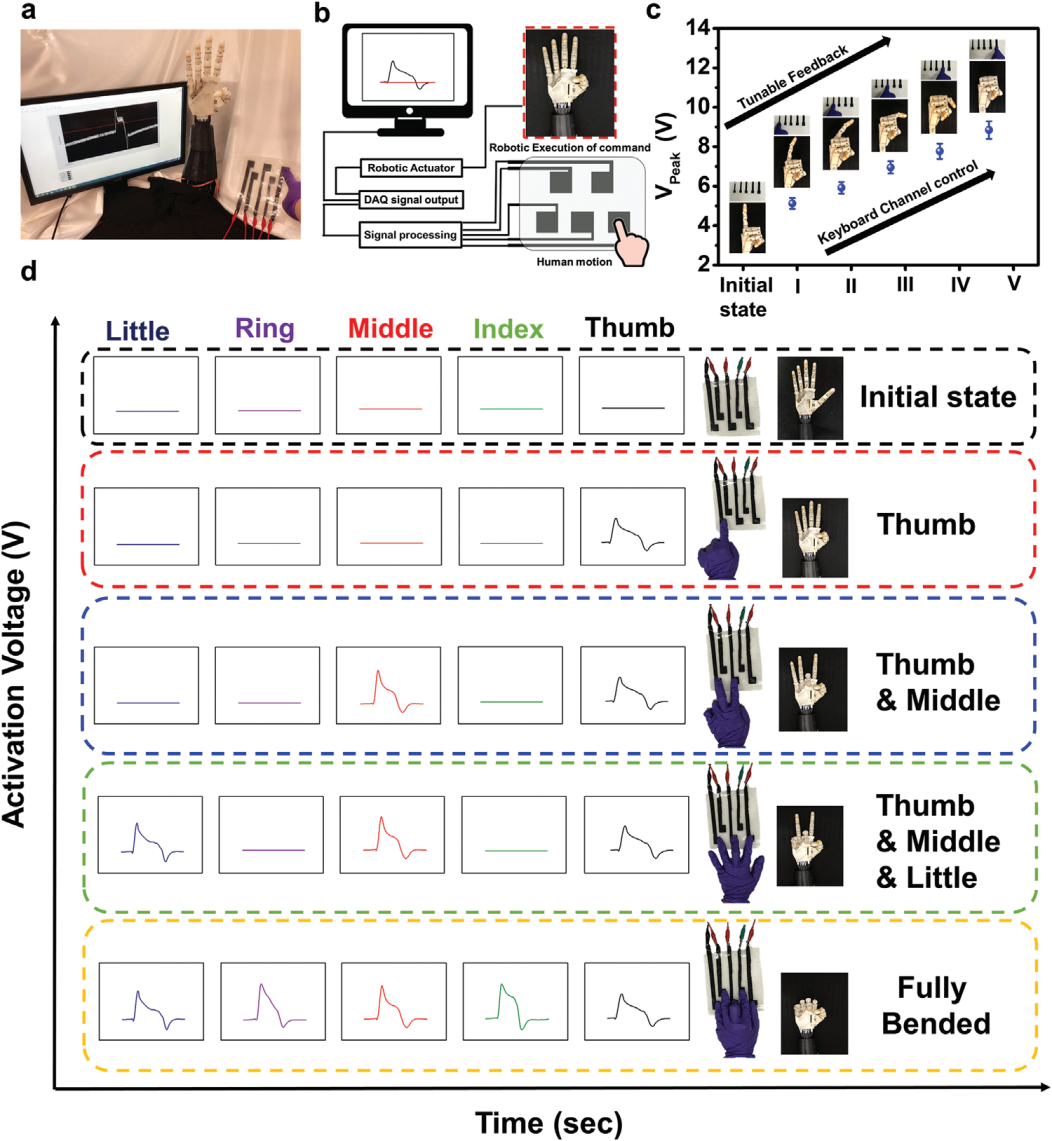
a) Photographs of a smart robotic assistive platform design. b) A schematic diagram of a system setup and signal processing network. c) The output voltage generated from cells made by B‐TENG to let individual robotic finger perform different bending motions. d) Photographs of multimode human–robotic interaction platform designed with B‐TENGs.

TENGs for human–machine interactive systems have been reported in various kinds of aspects, such as smart gloves, human–machine interface, and robotic skin.^[^ ^36^, ^37^, ^38^ ^]^ However, most of the previous attempts addressed the device/system development for healthy candidates. By considering clinical needs, especially for those who suffer from the situation with disability, they may not possess completed extremities to perform specific motions and desired activities. Herein, we demonstrate a smart assistive robotic platform by combining a robotic actuating system with a multifunctional keyboard based on B‐TENGs, as shown in **Figure** **4**a. The system setup and corresponding signal processing network are also illustrated in Figure 4b. When we touched the specific cell formed by B‐TENG, it can induce the pulse signals to be seen on the computing interface. Then the robot hands can be successfully driven to perform required gestures. In Figure 4c,d and Figures S10 and S11 in the Supporting Information, a multifunctional keyboard composed of five B‐TENG cells in different sizes was presented as a command interface to interact with the robotic actuating system. Before initiating the as‐designed platform, the channel definition on keyboard was assigned from “1” to “5” in Figure S11a in the Supporting Information. The force response of each cell was nearly similar (Figure S11b,c, Supporting Information). As shown in Figure 4c, the activation voltages generated by the finger tapping on different kinds of cell were measured. It is observed that different degrees of robotic finger bending (∆*θ* = 0°, 20°, 40°, 60°, 80°, 100°) can be effectively controlled by finger tapping on the specific size of cells. The bigger the cell size is, the larger the bending angle of robotic finger can be achieved. Furthermore, owing to the shape adaptability and tailorability of B‐TENG, another platform design by B‐TENG was presented, where five cells with identical sizes were integrated into a multichannel robotic assistive system. In Figure 4d, by tapping each of the cells, the activation of output voltages of different states including Initial state, Thumb, Thumb + Middle, Thumb + Middle + Little, and Fully bended state were displayed. The results indicate that as‐designed human‐robotic‐assistive platform possesses reliable feedback capability and selectivity. The real‐time demonstrations of human‐robotic platform are also shown in Video S1 and Video S2 in the Supporting Information. Moreover, this interactive platform can emulate the situation of candidates with disability to reach objects, as shown in Figure S12 and Video S3 in the Supporting Information.

**Figure 5:**
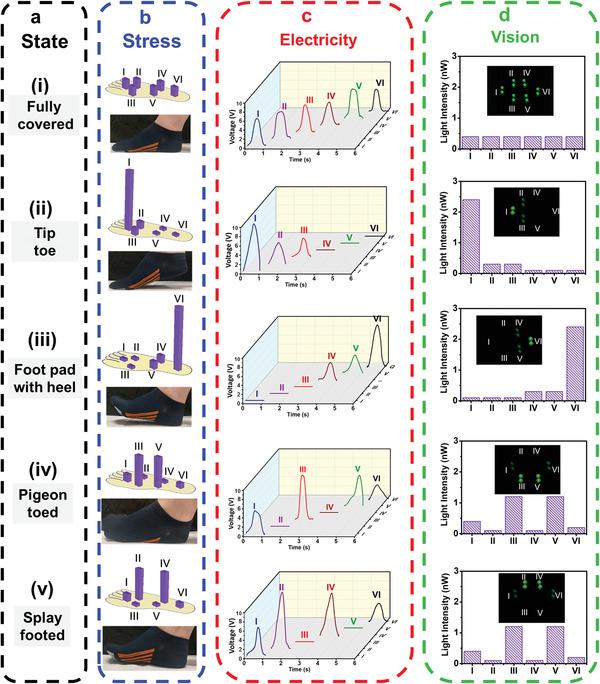
A self‐powered gait phase visualization platform. a) Various gait phase conditions assign for evaluation. b) Corresponded stress mapping profile of different gait phase conditions. c) Corresponded electrical signal distribution profile of different gait phase conditions. d) Corresponded optical power distribution of LED arrays for different gait phase conditions.

Gait phase identification incorporated with TENGs has been reported as a promising strategy to provide early detection of various kinds of chronic diseases, such as Alzheimer's and Parkinson's disease.^[^ ^39^ ^]^ Herein, the B‐TENG cells were embedded and distributed in the socks. There are five types of gait phase conditions for evaluation, which include fully covered, tiptoe, foot pad with heel, pigeon toe, and splay foot. **Figure** **5** demonstrates the gait phase detection with three‐step energy conversion and signal transition process. First, stress mapping data for each gait phase condition were collected by a commercial pressure sensor, as shown in Figure 5a. One can see that the distribution of stress mapping profile is consistent with the gait phase as designated. Moreover, owing to the self‐powered capability of B‐TENG, the output electrical signal can be clearly distinguished and monitored in real‐time with different gait phase conditions, as depicted in Figure 5b. More importantly, to visualize the detection signal, the socks with B‐TENG cells were connected to commercial LED arrays, as shown in Figure 5c. The LED arrays contain 12 LEDs, where two LEDs as a pair represent a position displaying the corresponded pattern of the gait phase. The light intensity of each pair was measured by a commercial optical detector. It can be observed that the optical mapping profile of LED arrays was consistent with both stress and electrical mapping results. The LED arrays can directly reflect the localized change of gait phase alteration that indicates the feasibility of using B‐TENG to be a self‐powered and wearable gait phase visualization platform. The real‐time monitoring demonstration is also provided in Video S4 in the Supporting Information.

**Figure 6:**
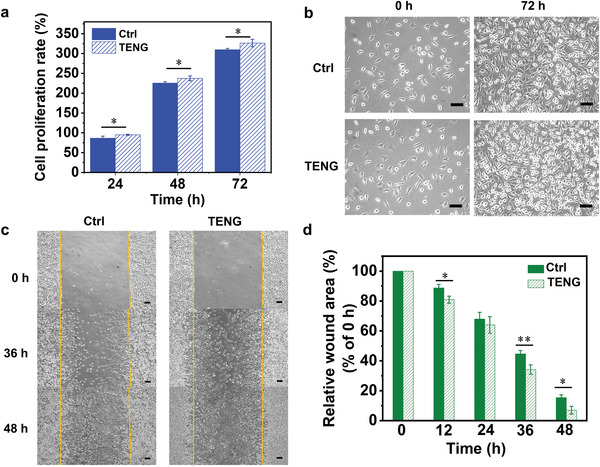
In vitro cellular behaviors of L929 fibroblasts by TENG stimulations. a) Proliferation rate of L929 cells stimulated by TENG and control cells in 24, 48, and 72 h (*n* = 3, *: *p* < 0.05, two‐tailed *t*‐test). b) Cell morphology at 0 and 72 h without (Ctrl) and with (TENG) electrical stimulation. The magnification is 100x and the scale bar is 100 µm. c) Scratched areas of the L929 fibroblasts in the control and the TENG stimulation groups at 0, 36, and 48 h. The magnification is 40x and the scale bar is 200 µm. d) Quantitative analysis of the migration results analyzed by ImageJ (*n* = 3, *: *p* < 0.05; **: *p* < 0.01, two‐tailed *t*‐test).

The combination of ES and TENG system has been recently treated as a promising approach in rehabilitation therapy, especially for wound healing‐related applications.^[^ ^40^, ^41^ ^]^ ES for wound healing simulates the natural wound‐healing mechanism of the endogenous electric field to promote skin growth.^[^ ^25^ ^]^ Cell proliferation and migration are one of the key indexes in evaluating biological activities within wound healing process. It has been reported that cell proliferation of L929 fibroblasts is effectively promoted under the ES by TENG within 10–50 µA.^[^ ^42^ ^]^ The proliferation‐related gene of L929 cells‐proliferating cell nuclear antigen (Pcna) was also found to be regulated after ES. Herein, to evaluate the feasibility of using B‐TENG as a suitable candidate to provide ES, the cellular behaviors of L929 fibroblasts were investigated by conducting cell proliferation and cell migration assay with stimulation by B‐TENG. Cells without ES were set as control groups. The proliferation rate of L929 fibroblasts was studied by comparing the cell viability cultured with and without ES, as shown in **Figure** **6**a. The proliferation rate of control and stimulated cells exhibited statistical significance at 24, 48, and 72 h. At 72 h, it can be observed that the proliferation rate of stimulated cells was 326.52 ± 9.35% which is higher than that of the control cells (309.58 ± 3.40%). The cell morphology of control and stimulated cells is also shown in Figure 6b demonstrating that the cell proliferation behavior with ES is promoted in comparison with the control group. As discussed above, the results indicate that the cell proliferation rate is enhanced by ES from B‐TENG. Figure 6c,d demonstrates the migration behavior of L929 fibroblasts stimulated by B‐TENG. It can be found that cells in the stimulated group moved faster than those in the control group. The relative wound area between control and experimental groups exhibited statistical significance at 12, 36, and 48 h. At 36 h, the relative wound area of stimulated group was 34.16 ± 3.15%, while the control group was 44.64 ± 2.24%. The remaining wound areas at 48 h were ≈15.36 ± 1.95% and 6.93 ± 2.61% of the initial areas in the control and stimulated groups, respectively. As manifested by L929 fibroblasts, the cell migration behavior can be enhanced by in vitro ES from B‐TENG.

**Figure 7:**
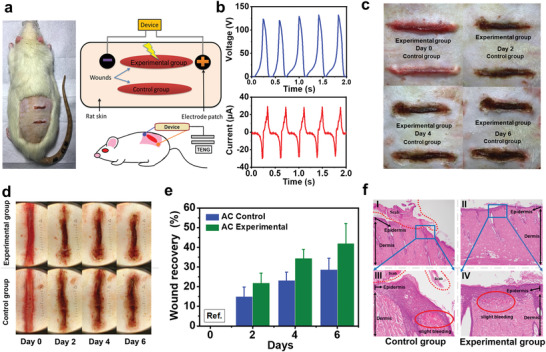
a) A schematic system setup by B‐TENG to induce ES for bruise therapy. b) The condition of output voltage and current of B‐TENG for ES. c) Digital images of the wound recovery process by inducing ES. d) Wound recovery images taken by dermoscopy. e) Changes in wound closure status. f) H&E‐stained tissue images of wounds 6 days after ES.

In addition to in vitro cellular behaviors, animal model test was further employed to confirm the feasibility of using B‐TENG in real wound healing applications. Herein, B‐TENG served as a self‐powered therapeutic unit to treat the as‐designed wound, where the wound recovery was statistically evaluated by the animal model. **Figure** **7**a shows the schematic system set up in an animal model, where the B‐TENG was applied to provide in vitro ES via external mechanical stimuli. The control and experimental groups of the wounds were located in a parallel configuration. Details of the wound creation are described in the Experimental Section. The output performance of B‐TENG was fixed at 30 µA current and 125 V voltage within the measurement, respectively, as shown in Figure 7b.

Dermoscopy has been widely used to record the localized changes in wound healing process, especially for the microstructure characterization of the epidermis, epidermal‐dermal junction, and dermis.^[^ ^43^ ^]^ In Figure 7c,d, photographs and dermoscopic images, which record the changes in the wound during continuous ES treatment are presented. It is observed that the wound length was shorter in experimental group as compared to the control group (Figure 7c). In Figure 7d, both control and experimental groups were characterized by dermoscopy. As can be seen on day 0, the vessels and tissues were damaged into a specific area by swelling, where the scabs are gradually formed from day 2 to day 6. It can be observed that the area of wound in the experimental group were significantly smaller than those in the control group. In addition, to quantify the rate of wound recovery, the changes of wound area were estimated from day 0 to day 6, as shown in Figure 7e. The detailed estimation process was also shown in Figure S13 in the Supporting Information. The results indicate that the wound shrinkage of the experimental group was higher than that in control group by day 2, day 4, and day 6. Furthermore, to understand the status of the wounds after ES treatment for 6 days, the tissues were sectioned by hematoxylin and eosin (H&E) staining, as shown in Figure 7f. Histological images at 20x (I, III) and 100x (II, IV) magnification of the control group and experimental group after ES treatments for 6 days are displayed. The histological image before the healing process is also shown in Figure S14 in the Supporting Information. It can be found that the epidermal wound of the experimental group has been re‐epithelialized. There is no obvious inflammation in the dermis, and only part of the dermis is connected to the epidermis with minor bleeding. More importantly, it can be observed that the wound surface of control group was covered with a layer of scab (Figure 7f‐I,III). The dermal tissue underneath has not been completely repaired. Cell proliferation and migration can be found in the vicinity of epidermal tissues. Slight bleeding can also be seen under the area of hyperplasia. As discussed above, it can be confirmed that the wound healing status after 6 days in experimental group (under ES) is better than that in control group.

## 3 Conclusion

In summary, a borophene/ecoflex nanocomposite combined with functional fabric as new triboelectric material is successfully demonstrated. The as‐designed borophene/ecoflex nanocomposite is flexible, cost‐effective, tailorable, and highly durable to various severe treatments. The as‐fabricated nanocomposite is further integrated into a B‐TENG with optimal dispersion condition of borophene NSs. The interfacial triboelectricity of borophene/ecoflex nanocomposite is also investigated by KPFM to access the origin of output enhancement in B‐TENG. The typical output voltage and current from the B‐TENG can reach 120 V and 20 µA, which were three times higher than those of the pristine device, respectively. The B‐TENG can deliver an instantaneous maximum output peak power density of 18 W m^−2^ at a matching load resistance of 20 MΩ that is able to drive the commercial LEDs and capacitors. Moreover, B‐TENGs are designed into multifunctional medical‐assistive and sensing platforms, where a smart human–robotic interface for upper limb assistive and gait phase detection for lower limb is demonstrated. Furthermore, B‐TENG serves as an in vitro ES device for wound therapy application. The in vitro cellular behavior and animal model observations were implemented to evaluate the device's feasibility. It is expected that the as‐designed B‐TENG will not only manifest the progress of self‐powered healthcare sensors, but also benefit the future technological applications from assistive technology to wearable therapeutics.

## 4 Experimental Section

### Synthesis of Borophene/Ecoflex Nanocomposite

The bulk borophene was ground into powder by grinding bowl, and subsequently dispensed into the acetone solution. The sample was then treated with an ultrasonic oscillator for 15 h at a constant temperature of 25 °C to produce borophene NSs. Afterward, the suspension solution was centrifuged to collect the supernatant. The supernatants with different volumes of borophene NSs were then prepared into mixing with ecoflex. Finally, the mixed solution was heated at 60 °C for 1 h to form nanocomposite layer with various concentration of borophene NSs.

### Device Fabrication of B‐TENG

The commercial carbon cloth was cut into size of (15 mm x 15 mm x 0.5 mm), and combined with the borophene/ecoflex nanocomposite previously prepared to serve as a conducting electrode. The whole nanocomposite layer was then regarded as a new negative triboelectric layer baked in an oven at 60 °C for 1 h. Finally, the polyester as a positive triboelectric layer was introduced to be integrated with the nanocomposite layer to produce a B‐TENG device.

### Materials Characterization

An optical microscope (Olympus BX51M) equipped with a charge‐coupled device (Leica DFC495) was used for optical imaging. The surface topography, material morphology, and elemental analysis of samples were conducted by AFM (Bruker Dimension Icon), SEM (JEOL JSM‐7800F), and EDS (Oxford systems), respectively. XPS spectra were measured with an ESCA spectrometer (Escalab 250, VG Scientific) coupled with a monochromatic X‐ray source (1486.6 eV Al Ka) and processed using CASA XPS software (v.2.3.17, Casa Software Ltd.). The XPS spectra were calibrated against the binding energy of the C 1s signal at 284.60 eV of the adventitious carbon prior to the spectral measurements, which was also used as the reference energy to correct the background charging effect. The spectral background subtraction was made using the Shirley–Sherwood method. The quantitative elemental compositions were calculated by integrating the highest peak intensity and considering the atomic sensitivity factor of each element. HR‐TEM (Technai G2 F20 FEG‐TEM) operating at 300 kV was used to examine the crystal structures, of which the data were analyzed using Digital Micrograph software. KPFM measurements were conducted by an AFM (Bruker Dimension Icon).

### Output Performance Characterization

The triboelectric outputs of B‐TENG were measured with a programmable electrometer (Keithley, Model 6514, 200 TΩ input impedance). A commercial linear motor system was applied to provide adjustable external force input. The voltage signals generated in B‐TENG sensors were collected and dealt with the homemade five channel system. Regarding the gait phase sensor, the analysis result of the stress signal was controlled by a digital pressure sensor (FP‐BTA).

### Artificial Robotic Components

The homemade 3D printed robotic hand was produced by a commercial 3D printer from XYZ Printing Technology. The well‐known fused filament fabrication (FFF) technology was executed with the main printing material of polylactide (PLA) at 210 °C. The source of the printed model comes from the Parloma project.

### Cell Culture

The L929 fibroblast cell line was purchased from Bioresource Collection and Research Center (BCRC, Hsinchu City, Taiwan). Cells were maintained in Dulbecco's modified Eagle medium (DMEM, Corning, NY, USA) supplemented with 10% fetal bovine serum (FBS, Corning, NY, USA) and 1% antibiotic of penicillin–streptomycin solution (Corning, NY, USA) at 37 °C in a 5% CO_2_ incubator (BB15, Thermo Fisher Scientific, MA, USA).

### Cell Proliferation and Migration

To investigate the cell proliferation induced by the electric stimulation of B‐TENG, 1 × 10^5^ cells of L929 fibroblasts were seeded in 35 mm diameter culture dishes (Figure S19, Supporting Information) for 24 h. Cells were regularly stimulated for 1 h per day. Cell morphologies were obtained by using an inverted optical microscope (Olympus CK30). The cell proliferation rate was evaluated by Cell Counting Kit‐8 at 24, 48, and 72 h. Cell migration was characterized by evaluating an in vitro scratch assay. Cells were seeded in 35 mm diameter culture dishes (1 × 10^6^ cells per dish) and grown at 37 °C in 5% CO_2_ incubator overnight. Confluent cells were maintained in DMEM containing 5% FBS. A straight scratch was made by using a sterile 1000 µL tip before B‐TENG stimulation. Cells were regularly stimulated for 1 h per day. The scratched regions were recorded by an inverted optical microscope (Olympus CK30) and the areas were calculated by using the ImageJ software (NIH, Bethesda, MD, USA).

### Statistical Analysis

Each experiment was repeated three times. The cell proliferation rate and the relative wound area were expressed as mean ± standard deviation (SD). The statistical analysis was performed by using the SPSS software (version 27.0.1.0, IBM SPSS, IL, USA). All results were analyzed by the two‐tailed *t*‐test, where *p* < 0.05 was considered statistically significant.

### Wound Recovery Experiments

Male Sprague–Dawley rats, aged older than 12 weeks, were purchased from BioLASCO Taiwan Co., Ltd. and used according to the protocols approved by Taipei Medical University Institutional Animal Care and Use Committee (LAC‐101‐0243). The rats were anesthetized with isoflurane (>400 mg; 3%, 5 L min^−1^) gas. The hemostatic forceps were used to fix the skin around the first section of the lower back lumbar spine in a fixed manner and time (3 min). The pressure was applied to produce two single areas (upper limit of about 3 cm^2^) with a distance of 1–1.5 cm of the injured area (the epidermis and dermis were preserved). The hemostatic forceps were removed and waited for the skin to be flat. An alternating current (AC) which formed microcurrent was applied to the wound recovery experiment. The actual treatment was given 24 h after the wound was generated. After the rats were anesthetized, the microcurrent electrical stimulation treatment (30 min per day for 6 days) was performed at the same time.

## Conflict of Interest

The authors declare no conflict of interest.

## Supporting information

Supporting Information

Supplemental Video 1

Supplemental Video 2

Supplemental Video 3

Supplemental Video 4

## Data Availability

Research data are not shared.
