# Supplementary material for: A Facile, Fabric Compatible, and Flexible Borophene Nanocomposites for Self‐Powered Smart Assistive and Wound Healing Applications
Source: Adv Sci (Weinh). 2022 Jun 3;9(22):2201507. doi: 10.1002/advs.202201507 (PMC9353498; doi:10.1002/advs.202201507)
Supplement: Supplementary file 1 — Supporting Information [file ADVS-9-2201507-s004.pdf]

ADVANCED SCIENCE
Open Access

Supporting Information

for *Adv. Sci.*, DOI 10.1002/advs.202201507

A Facile, Fabric Compatible, and Flexible Borophene Nanocomposites for Self-Powered Smart Assistive and Wound Healing Applications

*Shuo-Wen Chen, Shih-Min Huang, Han-Song Wu, Wei-Pang Pan, Shih-Min Wei, Chih-Wei Peng, I-Chih Ni, Bayu Tri Murti, Meng-Lin Tsai, Chih-I Wu and Po-Kang Yang**

Supporting Information

A Facile, Fabric Compatible, and Flexible Borophene Nanocomposites for Self-Powered Smart Assistive and Wound Healing Applications

Shuo-Wen Chen, Shih-Min Huang, Han-Song Wu, Wei-Pang Pan, Shih-Min Wei, Chih-Wei Peng, I-Chih Ni, Bayu Tri Murti, Meng-Lin Tsai, Chih-I Wu, and Po-Kang Yang[*]

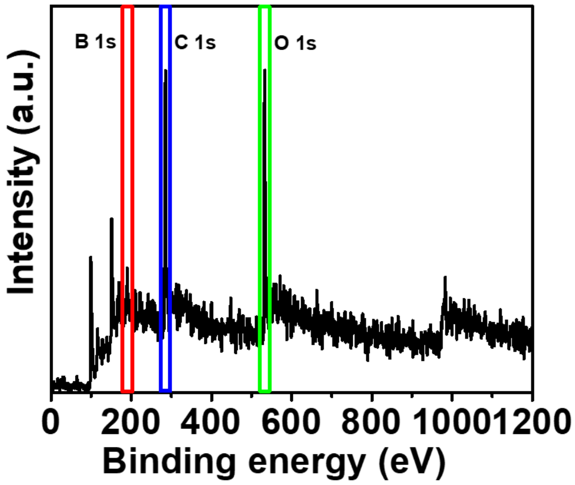

Figure S1. Wide-scan survey XPS spectrum of borophene NS.

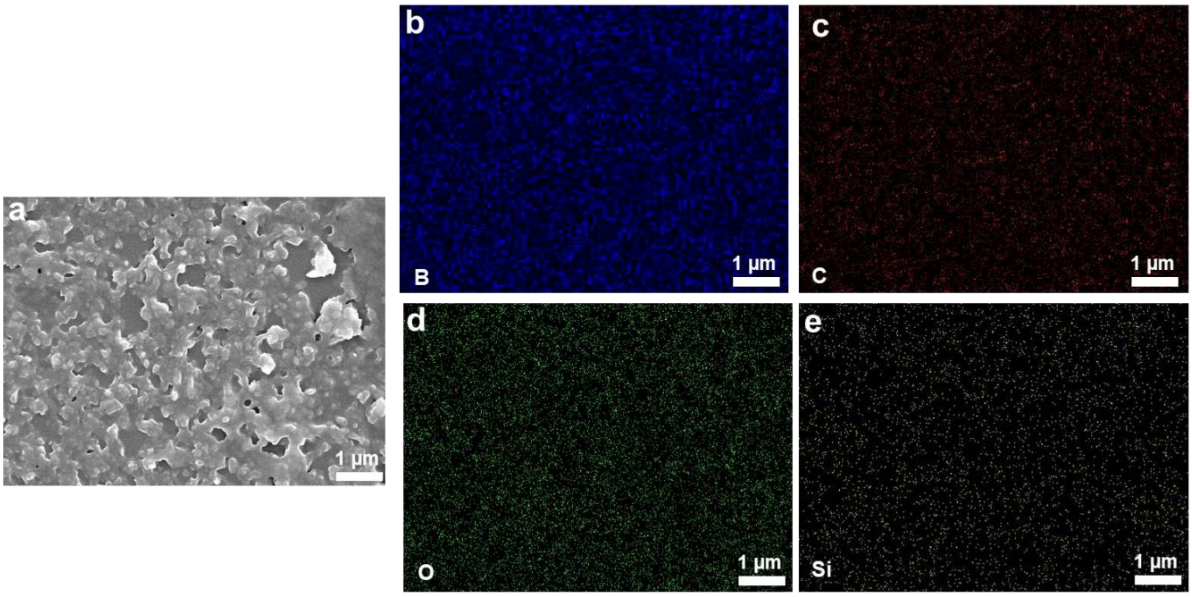

Figure S2. a) Surface morphology. b-e) EDS elemental mappings.

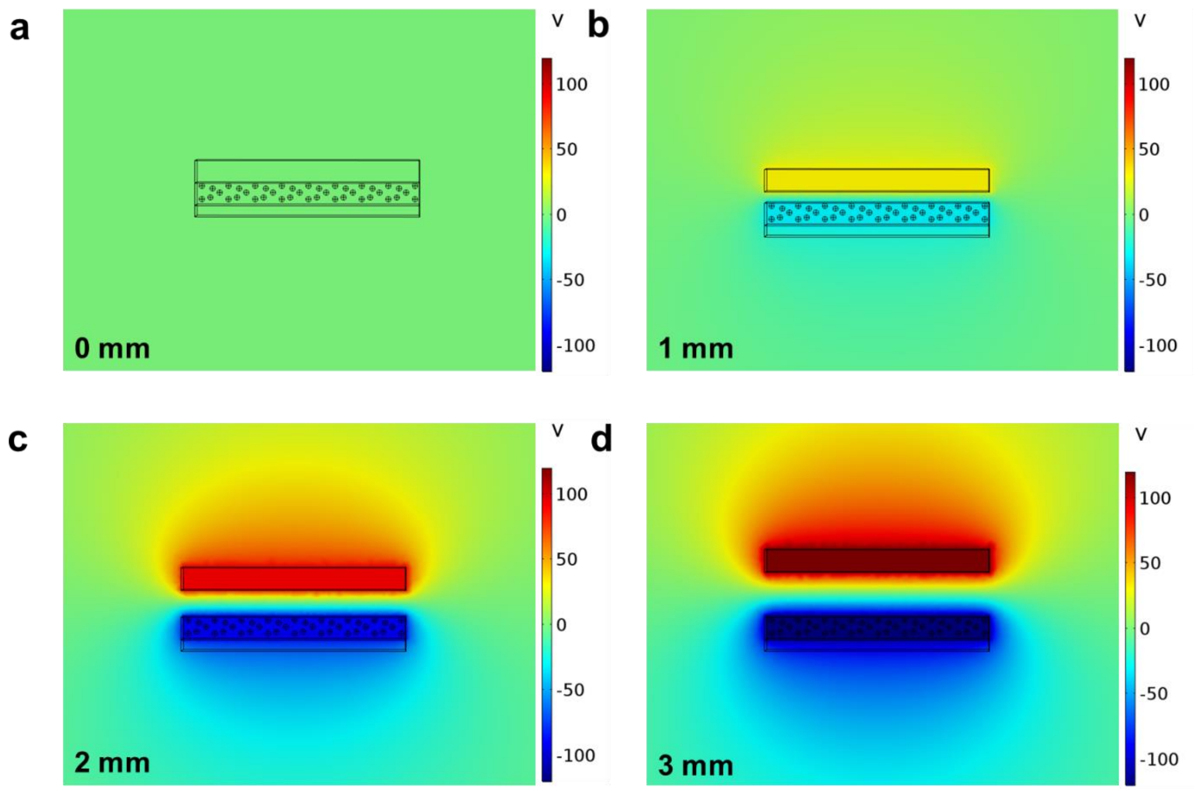

Figure S3. Simulated potential distributions of B-TENG within the contact-separation process i.e. a) 0 mm, b) 1 mm, c) 2 mm, and d) 3 mm.

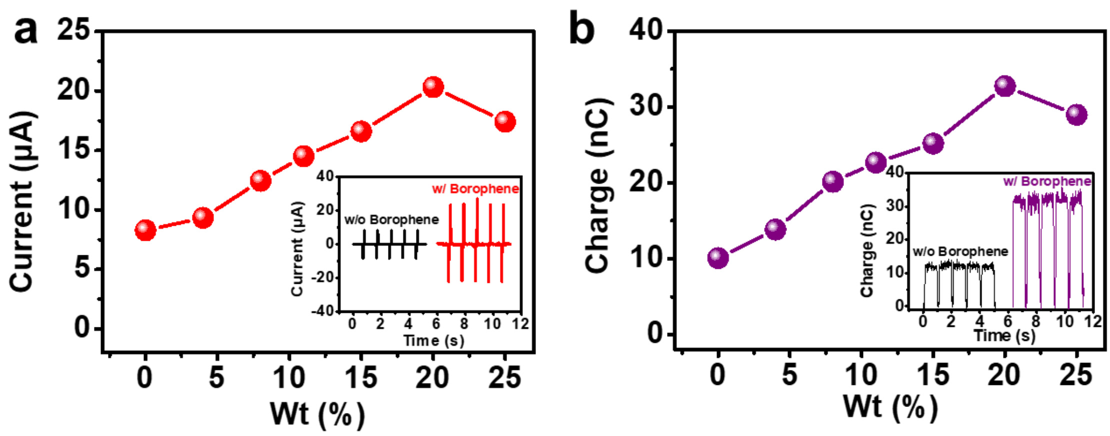

Figure S4. a) Output currents, and b) transferred charge characteristics of B-TENG with different borophene concentrations. The inset shows the output comparison between pristine device and B-TENG.

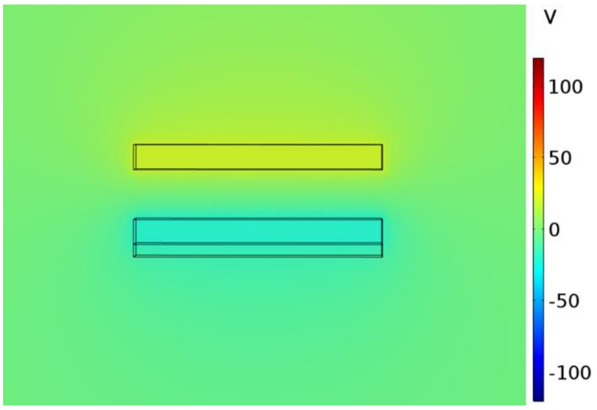

Figure S5. Simulated potential distribution between two tribo-interfaces inside the pristine device.

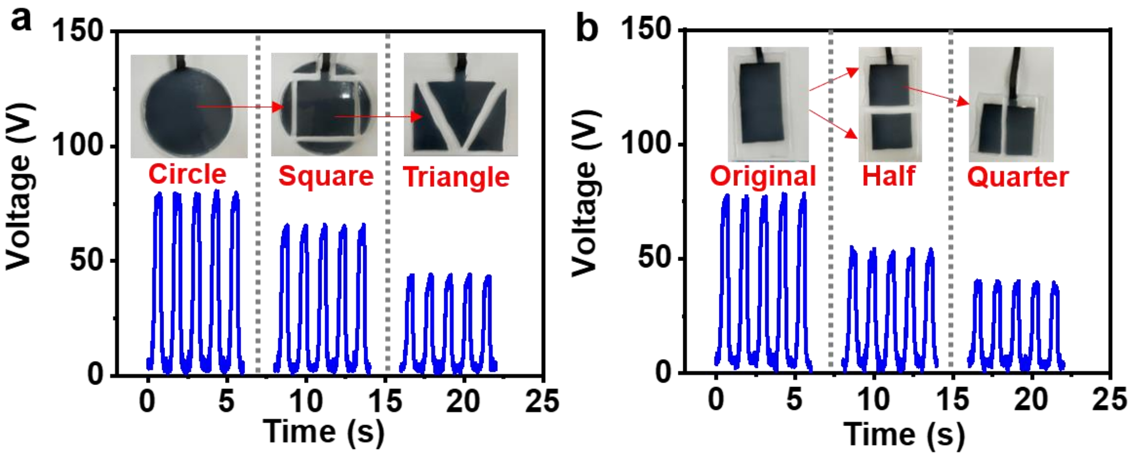

Figure S6. Tailorability of B-TENG in output voltages. a) Different shapes, and b) different sizes.

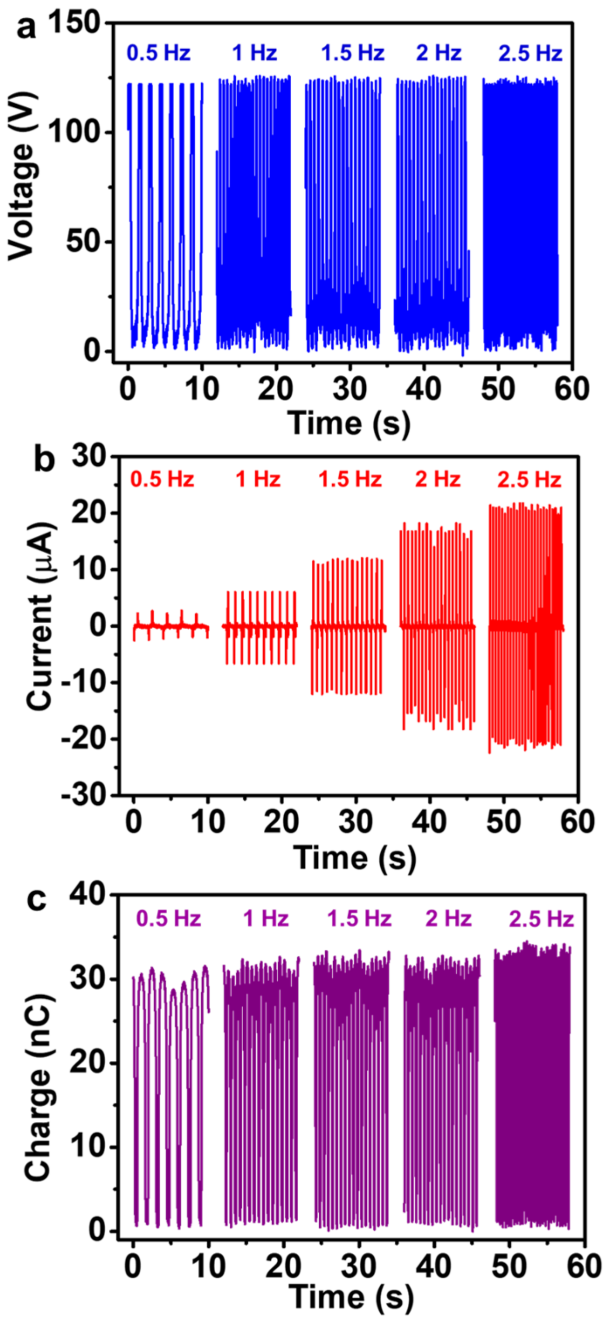

Figure S7. a) Output voltages, b) output currents, and c) transferred charges of B-TENG under different applied frequencies.

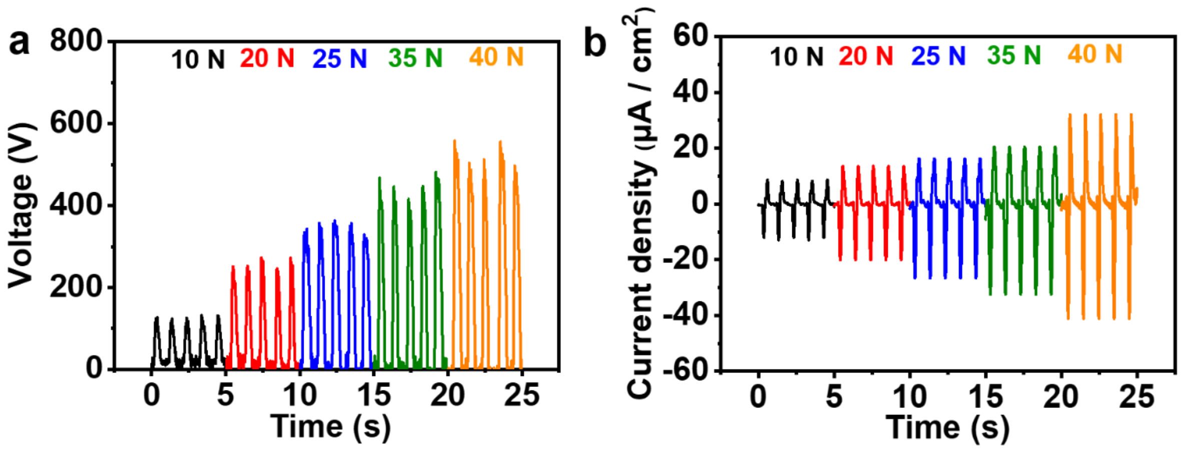

Figure S8. Output performance of B-TENG on applied pressure. a) Output voltages. b) Output current densities.

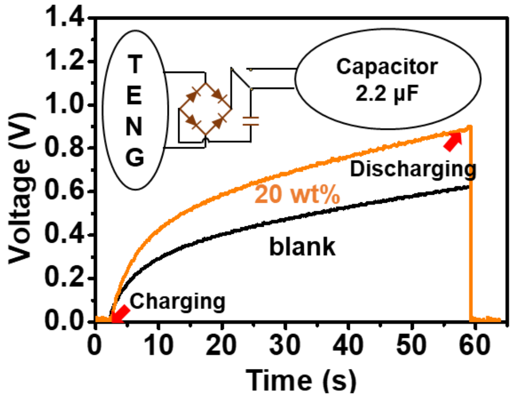

Figure S9. Stored charge-time relationship of the pristine device and B-TENG.

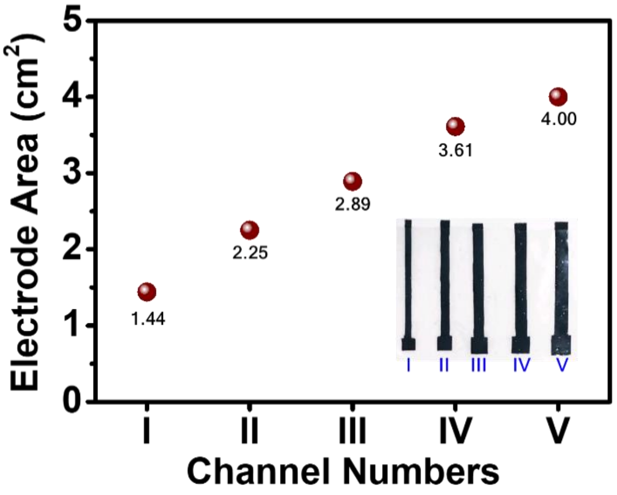

Figure S10. The smart keyboard composed of five B-TENG cells in different sizes. The inset shows the real sample photo of the five channel keyboard.

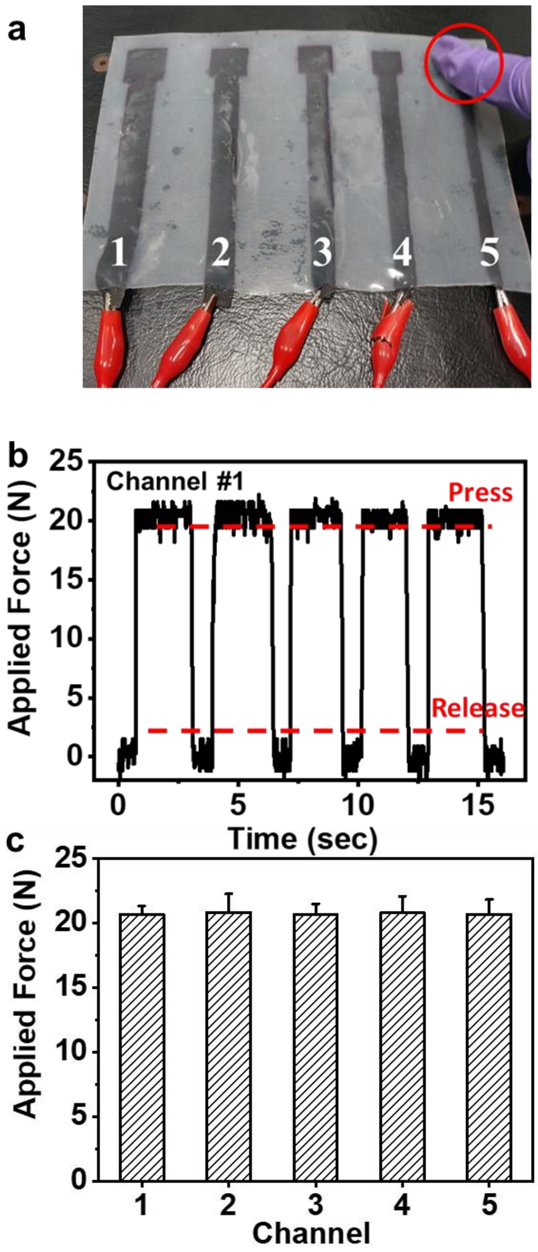

Figure S11. a) Five B-TENG cells with different sizes. b) The force response of channel#1 between pressing and releasing process. c) The force responses of different channels.

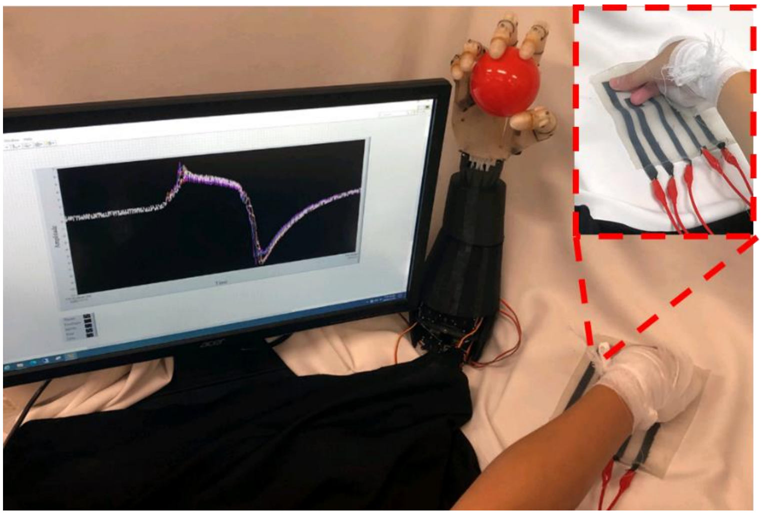

Figure S12. The emulation of the disabled candidate's situation by as-designed human-robotic platform.

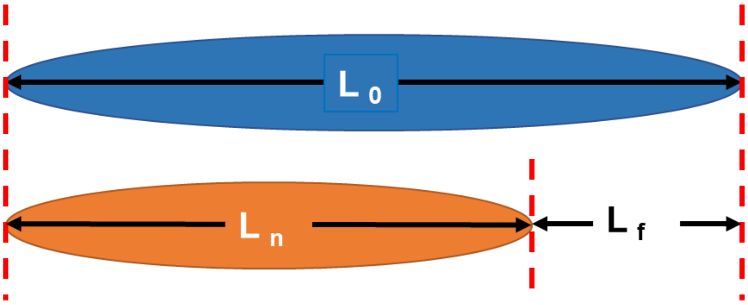

$$W : \frac{L_0 \text{ (cm)} - \mathbf{L_N} \text{ (cm)}}{L_0 \text{ (cm)}} \times 100\%$$

W : Wound closure (%)

L_0 : Wound length of Day_0 (cm)

L_n : Wound length of Day_n (cm)

L_f : Wound length of Day_0 (cm) $-$ Wound length of Day_n (cm)

Figure S13. The formula used to determine the recovery effect where L_0 is the length of the wound on day 0 and L_n is the length of the wound on day N. After subtracting each other, L_f is observed, and then divided by L_0. Finally, by multiplying with 100%, the healing effect of the wound on the N day is calculated.

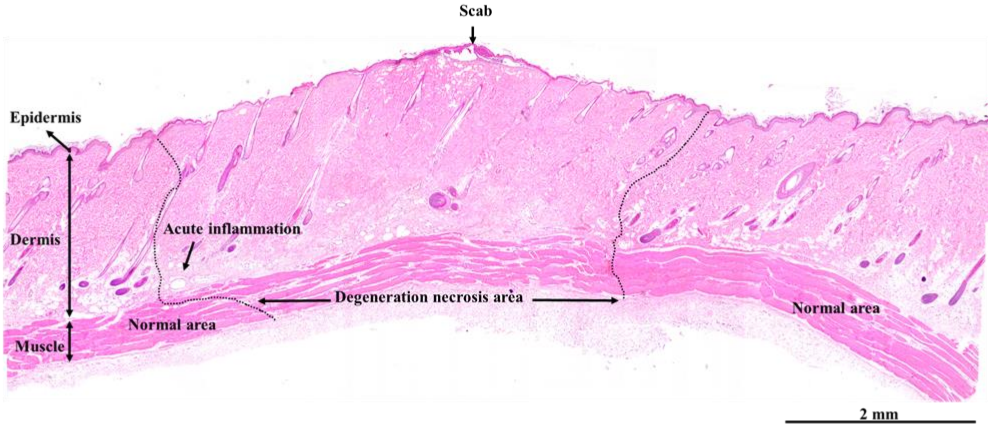

Figure S14. Histological image before wound healing.
